# Prolonged in vitro anti-bacterial, anti-inflammatory, and surfactant-promoting effects of volatile anesthetics

**DOI:** 10.1186/s12890-025-03849-w

**Published:** 2025-09-09

**Authors:** Claudia Scheffzük, Johanna Lührmann, Robin Brinkmann, Dominika Biedziak, Kristina Gloystein, Patrick Kellner, Cordula Stamme

**Affiliations:** 1 Division of Cellular Pneumology, Priority Area Infections, Research Center Borstel, Leibniz Lung Center, Borstel, 23845 Germany; 2 Department of Anesthesiology and Intensive Care Medicine, University Hospital of Schleswig-Holstein, Lübeck, 23538 Germany; 3 Department of Anesthesiology, Intensive Care and Pain Medicine, BG University Hospital Bergmannsheil Bochum gGmbH, Bochum, 44789 Germany; 4 Department of Internal Medicine, Berlin Jewish Hospital, Berlin, 13347 Germany; 5 Department of Internal Medicine, Segeberger Kliniken, Bad Segeberg, 23795 Germany; 6 Department of Vascular Surgery, DIAKO Krankenhaus gGmbH, Flensburg, 24939 Germany

**Keywords:** Volatile anesthetics, Sevoflurane, Desflurane, Cytokines, IL-8, ARDS, Pneumonia, *Pseudomonas aeruginosa*, A549 cells, Surfactant protein

## Abstract

**Background:**

Volatile anesthetics are gaining recognition for their benefits in long-term sedation of mechanically ventilated patients with bacterial pneumonia and acute respiratory distress syndrome. In addition to their sedative role, they also exhibit anti-bacterial and anti-inflammatory properties, though the mechanisms behind these effects remain only partially understood. In vitro studies examining the prolonged impact of volatile anesthetics on bacterial growth, inflammatory cytokine response, and surfactant proteins — key to maintaining lung homeostasis — are still lacking.

**Methods:**

Using an anaerobic chamber setup, we evaluated the effects of the most commonly used volatile anesthetics, Sevoflurane and Desflurane, at clinically relevant concentrations on the growth of *Pseudomonas aeruginosa*, *Escherichia coli*, and *Staphylococcus aureus*. Bacterial growth was monitored over 24 h, assessing OD_600_, CFU/ml, and growth rate during the *log phase*. In the same setup, but with aerobic conditions, we investigated the immunomodulatory properties of both anesthetics on human A549 cells, either with or without bacterial lipopolysaccharide (LPS, 1 µg/ml) stimulation. Over 48 h, we analyzed pro-inflammatory chemokine release using ELISA and assessed surfactant protein expression with Western blot analysis.

**Results:**

Sevoflurane and Desflurane significantly reduced *Pseudomonas aeruginosa* growth as expressed consistently in OD_600_ and CFU/ml starting after 12 h. Both volatile anesthetics also significantly reduced *Staphylococcus aureus* OD_600_ starting after 21 h. Sevoflurane (*p* < 0.01) and Desflurane (*p* < 0.001) counteracted LPS-induced interleukin-8 release by A549 cells after 48 h and significantly ( *p* < 0.01 and *p* < 0.05) enhanced the expression of the propeptide of surfactant protein C after 24 h.

**Conclusions:**

Prolonged anti-bacterial and anti-inflammatory effects of Sevoflurane and Desflurane include both the reduction of *Pseudomonas aeruginosa* and *Staphylococcus aureus* growth as well as the inhibition of LPS-induced chemokine release by A549 epithelial cells paralleled by an increase of surfactant protein expression. These effects highlight the potential of volatile anesthetics beyond sedation in supporting lung function in ventilated patients with respiratory failure.

**Supplementary Information:**

The online version contains supplementary material available at 10.1186/s12890-025-03849-w.

## Background

Bacterial pulmonal infections are a significant cause of prolonged mechanical ventilation in patients admitted to intensive care units (ICU) [1]. These infections may present either as a primary diagnosis upon admission or as complications arising during ICU treatment. Regardless of their onset, bacterial pulmonal infections in ICU patients are associated with high mortality rates, ranging between 20% and 40%, and place a considerable burden on global healthcare systems [2]. The bacterial pathogens responsible for hospital-acquired pneumonia and its subset, ventilator-associated pneumonia, are often polymicrobial in nature. Common pathogens in these settings include Gram-negative bacteria, such as *Pseudomonas aeruginosa*, *Klebsiella pneumoniae*,* Escherichia coli*, and Gram-positive bacteria, such as methicillin-sensitive and methicillin-resistant *Staphylococcus aureus* [3, 4].

Volatile anesthetics (VA) have gained recognition for their beneficial properties in the sedation of mechanically ventilated patients with bacterial pulmonal infections, particularly those with respiratory failure during acute respiratory distress syndrome (ARDS) [5]. Next to their sedative efficacy they may offer additional therapeutic benefits for ARDS patients: for example, in vitro and in vivo studies suggest that VA possess anti-bacterial and anti-inflammatory properties. Specifically, research has demonstrated that VA can directly affect bacterial growth, motility, and biofilm formation, though differences in study design — such as the choice of anesthetic, concentration, application method, and bacterial targets — complicate comparisons across studies [6–8]. ARDS patients often suffer not only from direct bacterial infection but also from sustained inflammation due to sepsis. In vivo ARDS models have shown that intrapulmonary administration of lipopolysaccharide (LPS), a key virulence factor of Gram-negative bacteria, can induce symptoms consistent with human ARDS [9, 10]. LPS has also been used in in vitro A549 cell cultures, which mimic the physiological characteristics of alveolar epithelial type II cells (AEC II). This setup has been used in previous studies as an in vitro model for ARDS [11, 12]. The anti-inflammatory [13, 14] and immunomodulatory [15] effects of VA have been observed in both in vitro [13, 16] and in vivo ARDS models [14, 17, 18], as well as clinical studies in ARDS patients [19, 20].

Pulmonary surfactant, a lipoprotein complex synthetized by AEC II cells, plays a crucial role in reducing surface tension at the air-liquid interface of the lungs, thereby facilitating proper gas exchange [21]. This biophysical function is primarily mediated by phospholipids and hydrophobic surfactant proteins (SP) [22]. However, mice lacking the hydrophobic surfactant protein SP-C, experience persistent inflammation following repeated LPS exposure and display intrinsic pulmonary inflammation along with heightened sensitivity to LPS [22, 23]. In addition, an intact LPS receptor (TLR4/CD14/MD2) is essential for SP-C inhibition of nuclear factor (NF)-kB-mediated expression [22].

In this in vitro study, we investigated the prolonged effects of two commonly used VA, Sevoflurane and Desflurane, on key pathophysiological aspects of ARDS. First, we examined the growth patterns of three bacterial strains frequently associated with pneumonia-related ARDS, *Pseudomonas aeruginosa (P. aeruginosa)*, *Escherichia coli (E. coli)*, and methicillin-sensitive *Staphylococcus aureus (S. aureus)*, employing VA dosages and administration modes that closely resemble clinical protocols. Second, we developed a prolonged cell culture model using human AEC II-derived A549 cells to assess the anti-inflammatory effects of VA. For this, A549 cells were continuously exposed to Sevoflurane or Desflurane, either with or without LPS, and chemokine production of interleukin (IL)−8 along with proSP-C protein expression were measured to assess the innate immune response.

## Materials and methods

### Bacterial culture and experimental set up

Three bacterial strains, commonly involved in hospital-acquired pneumonia, were analyzed (Detailed strain data derived from BacDive) [24]: Gram-negative bacterial strains, *Escherichia coli* 3B3BAP1 (BacDive ID 23001) and *Pseudomonas aeruginosa* PAO1 (BacDive ID 12801) [25], as well as the Gram-positive strain, *Staphylococcus aureus* SA113 (BacDive ID 14451).

All steps for bacterial cultivation were conducted under sterile conditions. Bacteria were cultured from stock in lysogenic broth in a CO_2_-enriched environment at 37 °C for 24 h. Following this, streaking was performed on agar plates to isolate colonies. After another 24 h, one colony-forming unit (CFU) was transferred into 5 ml of LB broth. After an incubation period of approximately 12 h, the optical density at 600 nm (OD_600_) was measured using photometric light-scatter analysis (Jenway 6320D Spectrophotometer, Cole-Parmer Ltd., Staffordshire, UK) and the bacterial suspension diluted to an OD of 0.1. Based on preliminary experiments (data not shown), the target concentration of the bacterial suspension was set at 100 CFU/ml. A total of 30 ml of this 100 CFU/ml bacterial suspension was placed in cell culture tubes for the subsequent experimental setup.

### A549 cell culture

The A549 cells used for the presented experiments were kindly made available by Prof. Dr. H. Heine, Research Center Borstel, Borstel, Germany. The human A549 lung cell line (ATCC CCL-185^TM^) has been first described by Giard et al. [26] and was further characterized by Lieber et al. [27] with respect to their surfactant protein production. Cultivation of A549 cells was performed according to the data sheet and previous experiments [28]. In short, freshly thawn cells were resuspended in RPMI culture medium (Roswell Park Memorial Institute culture medium, Pan-Biotech GmbH, Aidenbach, Germany) containing RPMI 1640, L-glutamine, 2.0 g/l NaHCO_3_, 10% fetal bovine serum and 1% penicillin-streptomycin. Resuspended cells were cultivated in a T25 cell culture flask (TC-Flask T25/T75, SARSTEDT AG & Co. KG, Nümbrecht, Germany) with an initial dose of 0.5 × 10^6^ cells in a humidified air (95%)/CO_2_ (5%) environment at 37 °C. After 5 days of culture period, adherent cells were detached from the plastic surface by 1x trypsin (Merck KGaA, Darmstadt, Germany), washed twice and transferred into a T75 cell culture flask for maintaining. For LPS exposure, LPS (1 µg/ml) of *E. coli* strain O55:B5 (Sigma-Aldrich GmbH) was used. The A549 cells were prepared in a 6-well plate (SARSTEDT AG & Co. KG) at a cell density of 1.5 × 10^5^/well (16600 cells/cm^3^) corresponding to the data sheet and preliminary experiments. At this point the concentration of fetal bovine serum in the RPMI culture medium was switched to 1%.

### Application of experimental gases

The application of experimental gases was performed in a custom-made, air-sealed anaerobic container (Oxoid™ Anaerobic Jar, Thermo Fisher Scientific Inc., Waltham, MA, USA, Fig. 1.B). Gas composition was monitored by a commercially available anesthesia monitor (Vamos, Dräger Medical GmbH, Lübeck, Germany, Fig. 1.A). Culture media were exposed to experimental gases for 24 h before incubation of cells to achieve state of equilibrium: 2.1–2.2% Sevoflurane (Sev, SEVOrane^®^ 100%, AbbVie GmbH & Co. KG, Wiesbaden, Germany, Fig. 1.A) or 6.0% Desflurane (Des, SUPRANE 100%, Baxter GmbH, Unterschleißheim, Germany, Fig. 1.A). Control gases (Ctrl) varied based on the type of culture: for bacterial cultures, an anaerobic environment was maintained (95% N_2_ + 5% CO_2_), while A549 cell cultures were kept in an aerobic environment (95% room air + 5% CO_2_, Fig. 1.A). Of note, briefly opening the culture flasks at each time point introduced small amounts of oxygen, preventing absolute anoxia in the bacterial culture setting. Bacterial cultures were held under constant shaking to achieve optimal VA penetration. In the case of A549 cells, for each cell culture experiment a triplet of A549 cells was exposed to either LPS or control (Fig. 1.B). Since cell harvesting of A549 cells was performed for each well independently, each well is treated as an independent sample.

**Fi. 1:**
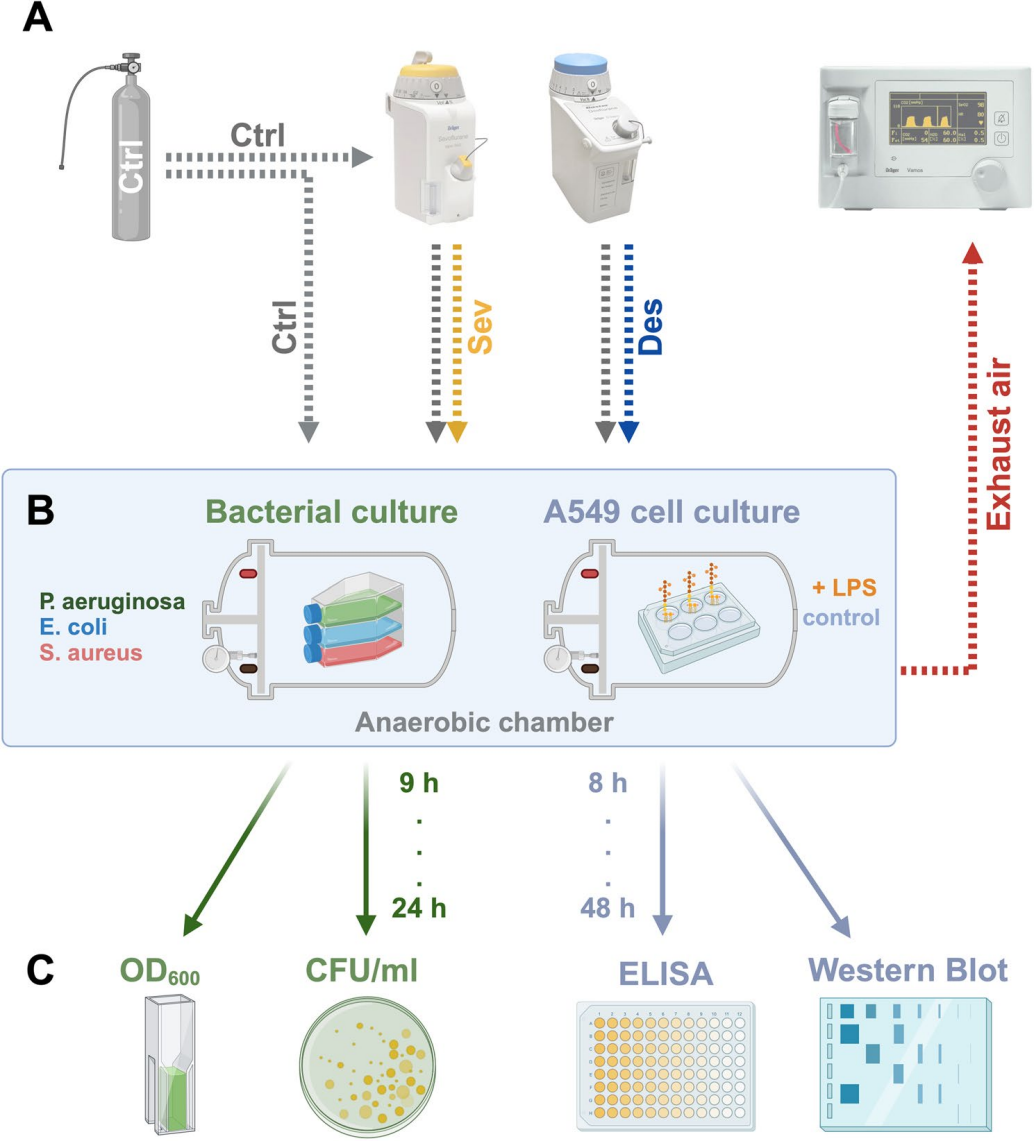
Model of experimental set up. (**A**) Control gases (Ctrl) varied based on the type of culture: for bacterial cultures, an anaerobic environment was maintained (95% N_2_ + 5% CO_2_), while A549 cell cultures were kept in an aerobic environment (95% room air + 5% CO_2_). The gas mixture also served as carrier gas for the application of two commonly used volatile anesthetics (VA). The concentration of Desflurane (Des, blue) and Sevoflurane (Sev, yellow) was measured by an external anesthesia gas detector which determined the concentration at the outgoing channel of gas conduction (exhaustion air, red). Gas concentrations were adjusted to MAC_50_, (2.1 to 2.2% for Sev and 6.0% for Des) within the anaerobic chamber. (**B**, l) Bacterial culture flasks with eft*Pseudomonas aeruginosa* (*P. aeruginosa*), *Escherichia coli* (*E. coli*), and *Staphylococcus aureus* (*S. aureus*) as well as (**B**, right) 6-well plates with A549 cell cultures were put into the anaerobic container after gas equilibrium was achieved. In the A549 cell culture 3 wells were additionally exposed to lipopolysaccharide (LPS) of *E. coli* (1 µg/ml). (**C**, left) Analysis of optical density at 600 nm wavelength (OD_600_) and colony forming units per milliliter bacterial suspension (CFU/ml) were performed after 9, 12, 15, 18, 21, and 24 h, respectively. (**B**, right) Analysis of Enzyme-linked Immunosorbent Assay (ELISA) and Western Blot analysis of pro-SP-C protein expression were performed 8, 16, 24, and 48 h after VA exposure

### Analysis

#### Bacterial growth

Sample harvesting was performed after 0, 9, 12, 15, 18, 21, and 24 h, respectively. For each of the time points 1.5 ml of bacterial solution were drawn from bacterial culture and analyzed. The analysis of changes in OD_600_ is regularly used as a surrogate parameter for bacterial growth measurements in liquid samples. Since cell density and OD_600_ tend to deviate at higher values (> 1), the bacterial suspension was diluted to target values of about 0.4 [29]. Since OD_600_ measures cannot distinguish between viable cells and debris leading to light-scattering, we used CFU counting per volume unit (CFU/ml) to determine the effect of VA on living bacteria. Bacterial growth can be subdivided into different phases depending on the progression of OD_600_ changes. The *log phase* describes the period of unrestrained cell division with sufficient substrate and negligible metabolic waste (Suppl. Figure 1.A). To determine the effect of VA on this crucial phase of bacterial cell division we compared the growth rate at the *log phase* under influence of both VA. Growth rate (µ) was calculated after determination of the exponential growth phase of each bacterial strain as described in Widdel 2007 [29, 30].

$$\:\mu\:\:=\frac{\text{ln}{OD}_{2}-\text{ln}{OD}_{1}}{({t}_{2}-\:{t}_{1})}$$

#### Cell viability testing

Sample harvesting for cell viability testing was accomplished after 8, 16, 24, and 48 h. The cell viability testing was performed by Erythrosin B staining (Sigma-Aldrich GmbH), distinguishing cells with intact (clear cells) from those with damaged plasma membrane (light red stain). Erythrosin B was added in a 1:10 ratio to the diluted cell suspension and incubated for 4 min at 4 °C. After manual cell count in a *Neubauer* hemocytometer (Paul Marienfeld GmbH & Co. KG, Lauda-Königshofen, Germany), viability was defined by the fraction of living cells compared to the total amount of cells (in %).

#### Enzyme-linked Immunosorbent Assay (ELISA)

Sample harvesting for Western blot analysis and ELISA was accomplished after 8, 16, 24, and 48 h. IL-8 was measured in the cell supernatant rather than intracellularly, and since its concentration at time point 0 - before stimulation and secretion begin - would be expected to be zero, this time point was not included in the analysis. The concentration of IL-8 in cell-free supernatants of A549 cells was quantified by ELISA (Invitrogen by Thermo Fisher Scientific Inc.) according to the manufacturer’s protocol. To point out the exclusive effect of VA on LPS-stimulated A549 cells, the difference in IL-8 concentration between LPS-stimulated and unstimulated cells after control gas exposure was calculated: IL-8 concentration under control conditions (without LPS stimulation) were subtracted from the IL-8 concentration with LPS stimulation and VA exposure (for each of the three groups: Ctrl + LPS, Sev + LPS and Des + LPS).

#### Sodium dodecyl sulfate polyacrylamide gel electrophoresis (SDS-PAGE) and Western blot analysis

Analysis of surfactant proteins was performed for SP-A and the propeptide of SP-C, pro-SP-C [31]. The expression of both surfactant proteins was determined by Western blot analysis of A549 cell lysates from each group and time point. Cell lysates were prepared by exposure to radioimmunoprecipitation buffer (TRIS-hydrochloride, NaCl, nonident P-40, sodium deoxcholate, SDS, with pH 8.0) for 30 min on ice and subsequent ultrasonication. Protein content was measured by the bicinchoninic acid reagent (Interchim S.A., Montluçon, France). 50 µg of the lysates were separated on SDS-PAGE and transferred to nitrocellulose membrane (Carl Roth GmbH & Co, KG, Karlsruhe, Germany). Membranes were incubated with anti-SP-A (rabbit polyclonal; 1:1000, Merck KGaA), anti-pro-SP-C (rabbit polyclonal; 1:1000, Merck KGaA) or anti-β-actin (mouse polyclonal, 1:50, Santa Cruz Biotechnology, Inc., Dallas, TX, USA).

Horseradish peroxidase-conjugated streptavidin mouse anti-rabbit IgG (Santa Cruz Biotechnology Inc., 1:2000) and horseradish peroxidase-conjugated streptavidin anti-mouse IgG (Cell Signaling Technology, Danvers, MA, USA; 1:2000) served as secondary antibodies. Semi-quantitative detection of surfactant proteins was achieved by electrochemiluminescence (Image Lab, BioRad Laboratories Inc., Hercules, CA, USA). Band intensities (arbitrary units, a.u.) were normalized to β-actin.

### Statistics

Statistical analysis was performed using Microsoft^®^ Excel for MacOS Sonoma, Version 16.89.1 (Microsoft Corp., Redmond, USA) and Prism^®^ 10 for Mac (GraphPad Software Inc., San Diego, USA). Normality was assessed using the Shapiro-Wilk test. For experiments involving repeated measures, a mixed-effects model was used to account for within-subject variability. Normally distributed data were analyzed using a Dunnett- and Šídák-corrected two-way ANOVA (α = 0.05). All data are presented as mean ± standard error of the mean (SEM). P values less than 0.05 were considered significant, with multiple asterisks indicating significance levels: Statistical significances are depicted as *p* < 0.05*, *p* < 0.01**, *p* < 0.001***. Asterisks denominate $ = Sevoflurane, # = Desflurane, + = Control. Color scheme in the figures depicts the following groups: grey = control, yellow = Sevoflurane, and blue = Desflurane. For experiments involving additional LPS stimulation, we further differentiate the groups: light brown = control + LPS, red = Sevoflurane + LPS, and violet = Desflurane + LPS.

Figure 1 (https://BioRender.com/t55n338) and Fig. 5 (https://BioRender.com/g79j733) have been created with BioRender.com.

## Results

### Effect of volatile anesthetics on bacterial measures

**Fig. 2:**
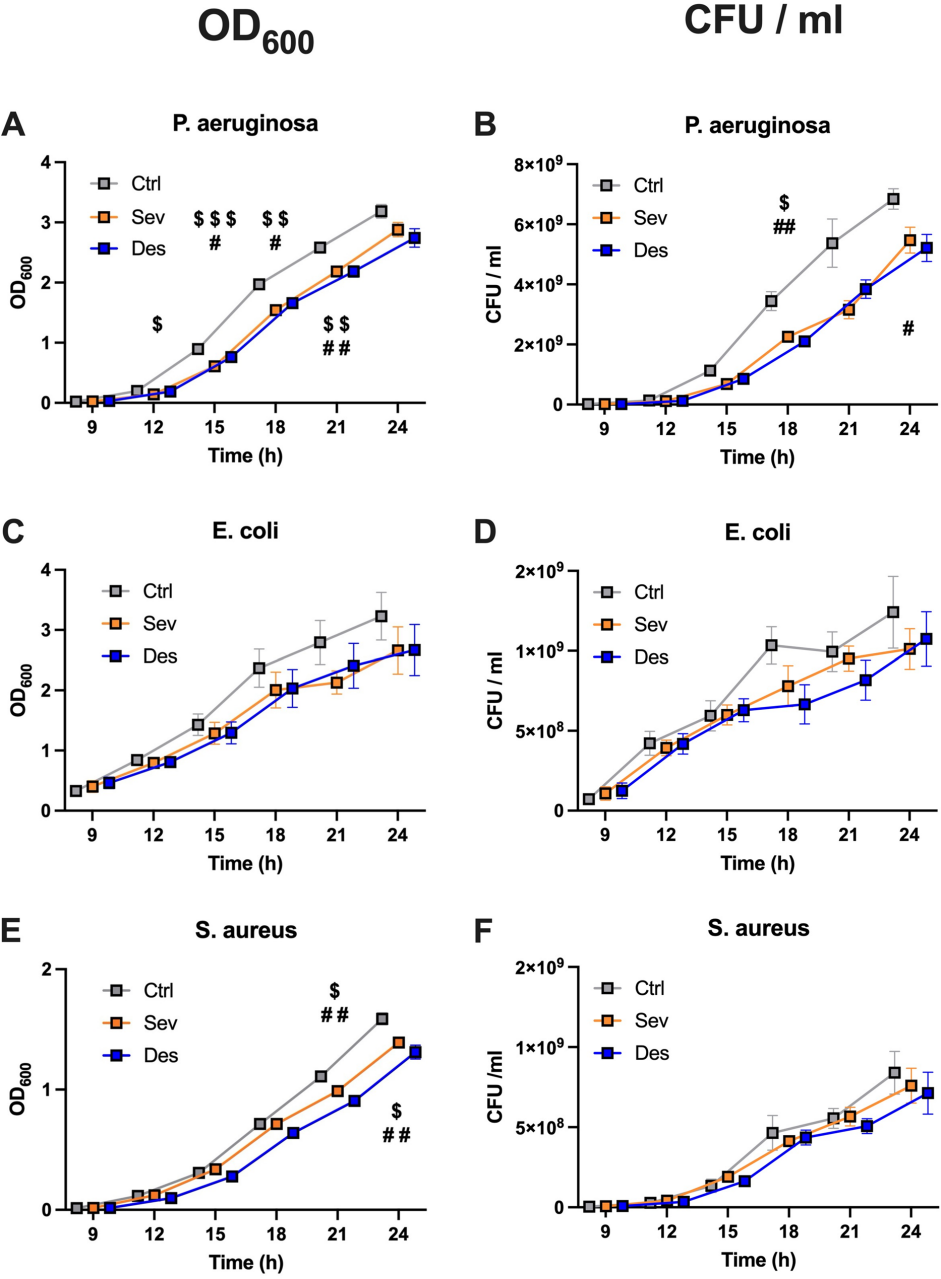
*Bacterial growth after exposure to* *volatile anesthetics* Measures of (**A**,** C**, **E**) adjusted optical density at 600 nm wavelength (OD_600_) and (**B**,** D**,** F**) colony forming units (CFU) per milliliter (ml) bacterial suspension are depicted for three bacterial strains associated with hospital-acquired pneumonia: two Gram-negative strains, *Pseudomonas aeruginosa* (*P. aeruginosa*) and *Escherichia coli* (*E. coli*), and one Gram-positive strain, *Staphylococcus aureus* (*S. aureus*). Data collection was performed 9, 12, 15, 18, 21, and 24 h after exposure to volatile anesthetics (Sevoflurane: yellow, Desflurane: blue) or Control gas (grey). Concentrations are depicted as CFU/ml. Ctrl: Control gas, Sev: Sevoflurane, Des: Desflurane. All results of are presented as mean +/- standard error of the mean (SEM) for *n* = 5–7. Statistical significances are depicted as *p* < 0.05 *, *p* < 0.01 **, *p* < 0.001 ***. Asterisks account for $ = Sev, # = Des

The three bacterial strains, *P. aeruginosa*, *E. coli*, and *S. aureus* were exposed to either control gas (Ctrl), Sevoflurane (Sev), or Desflurane (Des) for 9, 12, 15, 18, and 24 h. OD_600_ measurements indicated a gradual increase across all bacterial cultures. At 12, 15, 18, and 21 h, the application of Sevoflurane and Desflurane significantly reduced OD_600_ for *P. aeruginosa* compared to control conditions (12 h: *p* < 0.05 for Sev; 15 h: *p* < 0.001 for Sev and *p* < 0.05 for Des; 18 h: *p* < 0.01 for Sev and *p* < 0.05 for Des; 21 h: *p* < 0.01 for both Sev and Des; Fig. 2.A). At 21 and 24 h, also *S. aureus* cultures (21 h and 24 h: *p* < 0.05 for Sev and *p* < 0.01 for Des; Fig. 2.E) exhibited significantly reduced turbidity. A comparable trend for *E. coli* growth was detectable but did not reach significance (Fig. 2.C). The overall OD_600_ exhibited a continuous increase for all three bacteria.

Interestingly, the anti-bacterial effect of the CFU measures was consistent only for *P. aeruginosa* after 18 h (*p* < 0.05 for Sev and *p* < 0.01 for Des; Fig. 2.B) and 24 h (*p* < 0.05 for Des; Fig. 2.B). *E. coli* showed a slight trend after 18 h of exposure to both VA, but, again, did not reach significance (Fig. 2.D). *S. aureus* did not exhibit any noticeable effect (Fig. 2.F). When assessing growth rates during the *log* phase, none of the three bacterial strains showed any differences compared to control conditions, suggesting that a VA effect is unlikely (Suppl. Figure 1.B).

### Effect of volatile anesthetics on inflammation

**Fig. 3:**
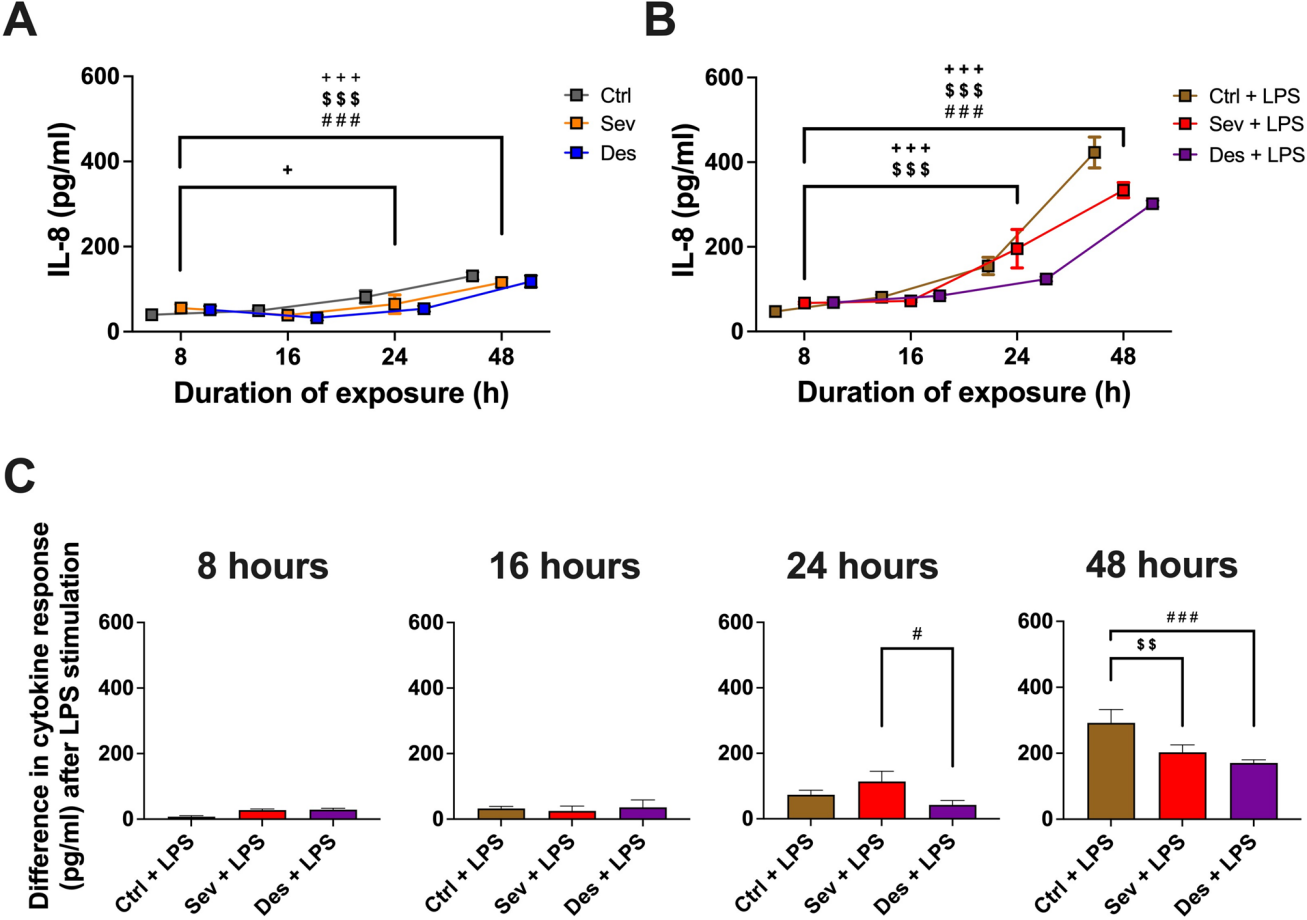
*Prolonged effects of volatile anesthetics on IL-8 release by A549 cells under basal and LPS-induced conditions* (**A**) Baseline interleukin (IL)-8 release in the presence of control gas (Ctrl; consisting of 95% room-air and 5% CO2), 6.0% Desflurane (Des), or 2.1-2.2% Sevoflurane (Sev). (**B**) IL-8 release in the presence of 1 µg/ml lipopolysaccharide (LPS) and Ctrl, Des, or Sev. (**C**) To assess, if volatile anesthetics (VA) exposure has an anti-inflammatory effect on LPS-exposed A549 cells, the difference in IL-8 concentration (pg/ml) between untreated and LPS-treated conditions was analyzed (for further details on the calculation method see results section). Measures were obtained throughout prolonged exposure (8, 16, 24, and 48 h) to VA. All results of are presented as mean +/- standard error of the mean (SEM) for *n* = 6 experiments. Statistical significances are depicted as *p* <0.05*, *p* <0.01**, *p* <0.001***. Asterisks account for + = Ctrl, $ = Sev, # = Des.

In our study, the IL-8 concentration measured after 24 and 48 h exhibited a constitutive increase of the neutrophil attractant, pro-inflammatory chemokine IL-8 over time (8 h vs. 24 h: Ctrl *p* < 0.05; 8 h vs. 48 h: Ctrl/Sev/Des *p* < 0.001) (Fig. 3.A). Under unstimulated conditions, a slight decrease in IL-8 concentration was detectable after 16 and 24 h, however, this effect was not considered significant (Fig. 3.A). In contrast, co-stimulation with LPS led to a significant boost in IL-8 concentration for all three groups (8 h vs. 24 h: Ctrl + LPS/Sev + LPS *p* < 0.001; 8 h vs. 48 h: Ctrl + LPS/Sev + LPS/Des + LPS *p* < 0.001) (Fig. 3.B).

When calculating the difference in IL-8 concentration between LPS-stimulated and unstimulated cells after control gas exposure (Fig. 3.C, details on the calculation see Methods), the anti-inflammatory effect of VA became apparent after 48 h. The strongest reduction in IL-8 concentration was discernible for Desflurane (Ctrl + LPS vs. Des + LPS: *p* < 0.001), followed by an equally significant reduction in IL-8 concentration after Sevoflurane exposure (Ctrl + LPS vs. Sev + LPS: *p* < 0.01). This was preceded by a smaller, but equally significant IL-8 decrease for Sevoflurane exposure after 24 h (Sev + LPS vs. Des + LPS: *p* < 0.05).

To account for potential bias from the toxic effects of VA, we conducted cell viability tests on A549 cells over a prolonged time of 48 h (Suppl. 2). The lowest cell viability was detected for Sevoflurane treatment (90% [87–96], averaged over all time points (8, 16, 24, and 48 h). Since more than 90% of the cells remained viable across all groups, a significant toxic effect of VA on A549 cells can be ruled out under the experimental conditions (Suppl. Figure 2.A). This result was also consistent in the LPS-treated group, despite the potential cytotoxicity of LPS on cell cultures. In this group, Sevoflurane combined with LPS showed the lowest viability (91,5% [88–93]; Suppl. Figure 2.B).

### Effect of volatile anesthetics on surfactant protein expression

**Fig. 4:**
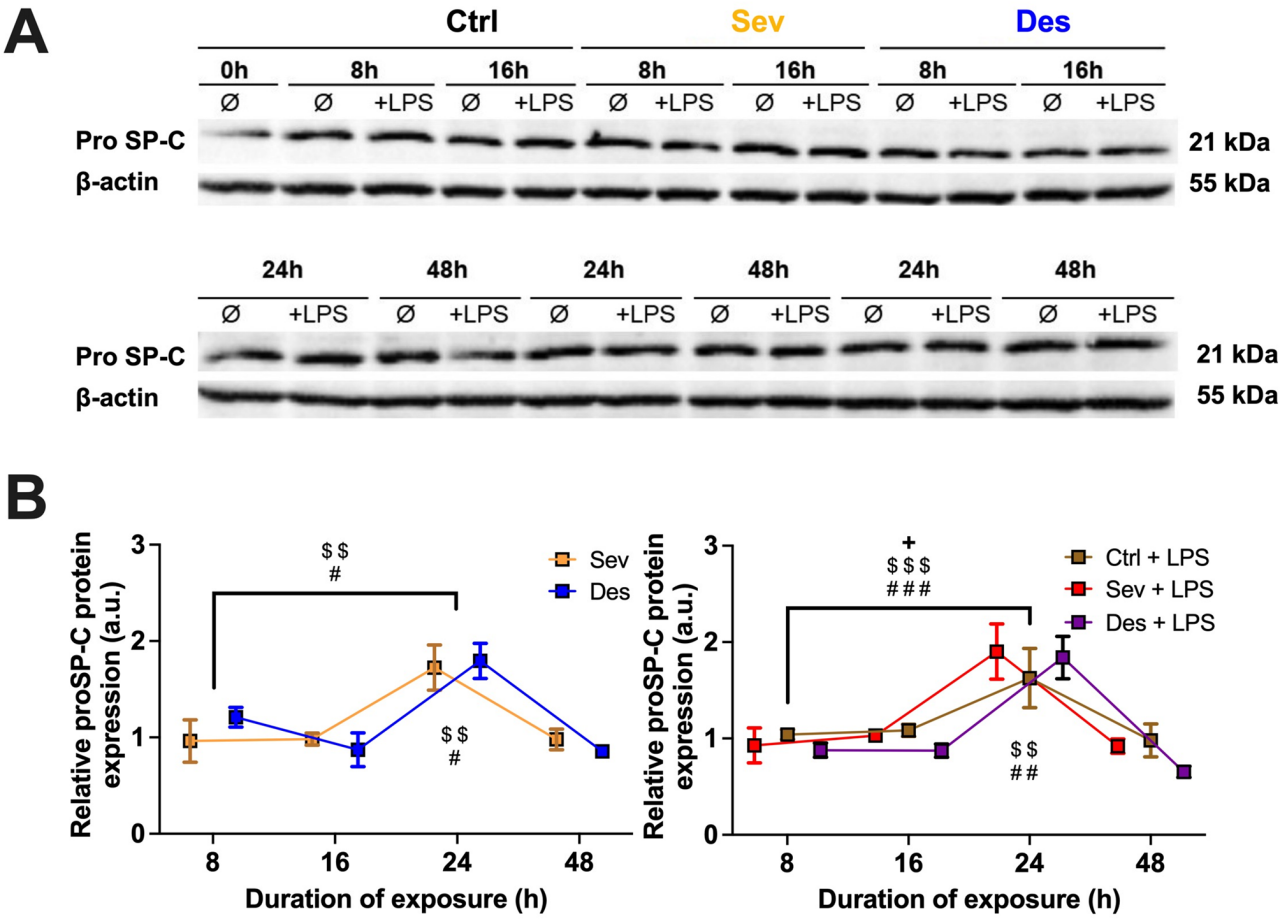
*Prolonged effects of* volatile anesthetics *on pro-SP-C expression by A549 cells under basal and LPS-induced conditions* A549 cells (1.5 × 10^5^ cells/well) were treated with control gas (Ctrl; consisting of 95% room-air and 5% CO_2_), 6.0% Desflurane (Des), or 2.1–2.2% Sevoflurane (Sev) in in the absence (**B**, left) or presence of 1 µg/ml lipopolysaccharide (LPS) (**B**, right) for the times indicated. Equal amounts of cell lysates were subjected to SDS-PAGE and immunoblotted for propeptide of surfactant protein C (pro-SP-C) and β-actin. (**A**) Full blots are provided in Supplement 4. For comparison, the results presented in arbitrary units (a.u.) were put into relation to those of Ctrl at each time point. Measures were obtained throughout prolonged exposure (8, 16, 24, and 48 h) to volatile anesthetics (VA). All results of are presented as mean +/- standard error of the mean (SEM) for *n* = 4–6. Statistical significances are depicted as *p* < 0.05*, *p* < 0.01**, *p* < 0.001***. Asterisks account for + = Ctrl, $ = Sev, # = Des

Exposure to VA led to a significant increase in relative protein expression of pro-SP-C after 24 h compared to baseline at 8 h. This effect was true for Sevoflurane (Fig. 4.B, left, *p* < 0.01) and Desflurane (Fig. 4.B, left, *p* < 0.05) without co-stimulation as well as all LPS-exposed groups (Fig. 4.B, right, Sev + LPS and Des + LPS *p* < 0.001, Ctrl + LPS *p* < 0.05). Comparing the group effect at 24 h to control condition (corresponds to relative proSP-C protein expression 1 a.u.), Sevoflurane or Desflurane exposure alone (Fig. 4.B, left, Ctrl vs. Sev *p* < 0.01, Ctrl vs. Des *p* < 0.05) achieved significant increase of pro-SP-C protein expression. LPS co-stimulation induced an even more prominent increase in relative pro-SP-C protein expression compared to Ctrl at 24 h (Fig. 4.B, right). The effect was significant for Sev + LPS and Des + LPS (Ctrl *p* < 0.01 each), but not for Ctrl + LPS under control gas conditions.

In contrast to the findings for pro-SP-C, SP-A showed only a minimal increase in relative protein expression following Desflurane exposure after LPS treatment compared to control conditions, while all other measurements showed no significant effect (Suppl. 3). Since SP-A is not membrane-bound, unlike pro-SP-C, and is instead secreted into the culture medium, it is possible that we only detected a small fraction of the total protein expressed due to prior excretion of SP-A, which may explain the lack of a significant effect. Based on this assumption, we conducted a qualitative control experiment (*n* = 2), further investigating the influence of VA on SP-A mRNA expression. Especially during the first 12 h, exposure to VA under both normal and LPS conditions resulted in a two-fold increase in SP-A mRNA expression compared to the control gas, with Sevoflurane showing a particularly pronounced effect. However, this effect diminished after 24 h, returning to baseline levels.

## Discussion

Volatile anesthetics are increasingly used for long-term sedation of critically ill patients on the ICU [32]. Recent evidence suggests that these sedative agents also exhibit anti-bacterial and anti-inflammatory properties and can exert positive effects on patients with ARDS, such as improved oxygenation, less epithelial injury and decreased cytokine levels [19]. To investigate whether VA directly affect bacterial growth, we designed an in vitro experiment involving three strains commonly associated with nosocomial bacterial pulmonal infections and exposed them to the most prevalent VA, Sevoflurane and Desflurane. Additionally, we examined the effects of these VA on human A549 cells under baseline conditions and after LPS stimulation, focusing on the pro-inflammatory cytokine IL-8 and the surfactant components pro-SP-C and SP-A. Both Sevoflurane and Desflurane significantly inhibited *P. aeruginosa* growth, as reflected by reductions in OD_600_ and CFU/ml. Similarly, they impaired *S. aureus* growth. In addition to their antibacterial effects, both anesthetics reduced LPS-induced IL-8 release in A549 cells and enhanced pro SP-C protein expression significantly, highlighting their potential role in modulating pulmonary inflammation.

Few studies have investigated the direct anti-bacterial effects of VA, typically using brief exposure durations, direct liquid application onto bacteria, or non-clinically relevant dosages [6–8]. Given the unique characteristics of an ARDS lung — such as protein-rich edema, an inflammatory environment, and pH shifts — experiments assessing the effects of VA on bacterial growth in an ARDS model must also take a possible oxygen-depletion into account. Consequently, in our bacterial experiments, we established an anaerobic environment using an anaerobic chamber filled with 95% N₂ and 5% CO₂ (Fig. 1). Both VA were administered at clinically recommended doses corresponding to MAC_50_ over a 24-hour period. The bacteria were inoculated in a defined quantity and maintained under continuous orbital shaking to ensure optimal penetration of VA into the medium.

The most notable finding from our experiments was the consistent and significant reduction in bacterial growth of the Gram-negative bacterium *P. aeruginosa*, as indicated by both OD_600_ and CFU measurements starting at 12 h. For *S. aureus* and *E. coli*, the results also showed a distinct trend in the same direction, albeit not as prominent.

It has been hypothesized that the lipophilic properties of VA have a more pronounced effect on Gram-negative bacteria compared to Gram-positive species, due to the latter’s well-defined murein lipid bilayer [7]. This effect is thought to be mediated by the fluorinated groups of VA, which may alter membrane permeability and fluidity [16]. Supporting this notion, a study by Martinez-Serrano et al. [7] demonstrated a stronger impact on the growth of Gram-negative bacteria compared to Gram-positive bacteria. However, the exact mechanism underlying the anti-bacterial effect of VA remains unclear. Bacterial growth is heavily influenced by environmental factors like pH, nutrients, and gas composition. The optimal time to assess unrestricted growth is during the *log* phase, before nutrient depletion, waste accumulation, or environmental changes affect proliferation. Interestingly, in our study, bacterial growth during the *log phase* was not affected by VA in any of the strains at any time point. Therefore, it is plausible to suspect that other factors contribute to growth impairment at later stages.

The ability to autoaggregate and form biofilms are key bacterial virulence factors, which are believed to be enhanced under hypoxic and anaerobic conditions, such as those present in ARDS and our experimental set up [33]. Autoaggregation has been observed in shaken cultures, where it initially increases turbidity and subsequently raises OD_600_ values [34]. However, in the later stages of autoaggregation, precipitates concentrate at the bottom, decreasing turbidity and OD_600_ values. A similar effect was observed in our experiments around 29 h, despite the continuous shaking of the bacterial media (results not depicted). All three strains — *P. aeruginosa*, *E. coli*, and *S. aureus* — are recognized for their capacity to form biofilms, particularly the *Pseudomonas spp.* in the context of ventilator-associated pneumonia [35–37]. Since we could not identify a direct bactericidal activity affecting bacterial growth, it is possible that the observed changes in OD_600_ measurements result from direct effects of VA on bacterial autoaggregation and biofilm formation. Chamberlain et al. [6] tested this hypothesis using the VA Isoflurane and Sevoflurane and surprisingly found an increase in biofilm formation for the same bacterial strains. They suggested that a possible mechanism of action involves an interaction between VA and species-specific ion transporters. This finding contrasts with the results of our experiments, where we propose an inhibitory effect of VA on bacterial aggregation. However, there are three significant methodological differences to consider: (I) Unlike our experiments, they facilitated biofilm formation by using 96-well plates, thereby creating a high-surface-low-volume environment. (II) The bacterial cultures in their study were not shaken. (III) Anoxic conditions have shown to upregulate virulence factors in facultative anaerobic bacteria like *P. aeruginosa* [38] and *S. aureus* [39].These differences may result in differential effects of VA on bacterial autoaggregation and motility. Emerging antibiofilm assessment methods could also help validate our findings [40].

**Fig. 5:**
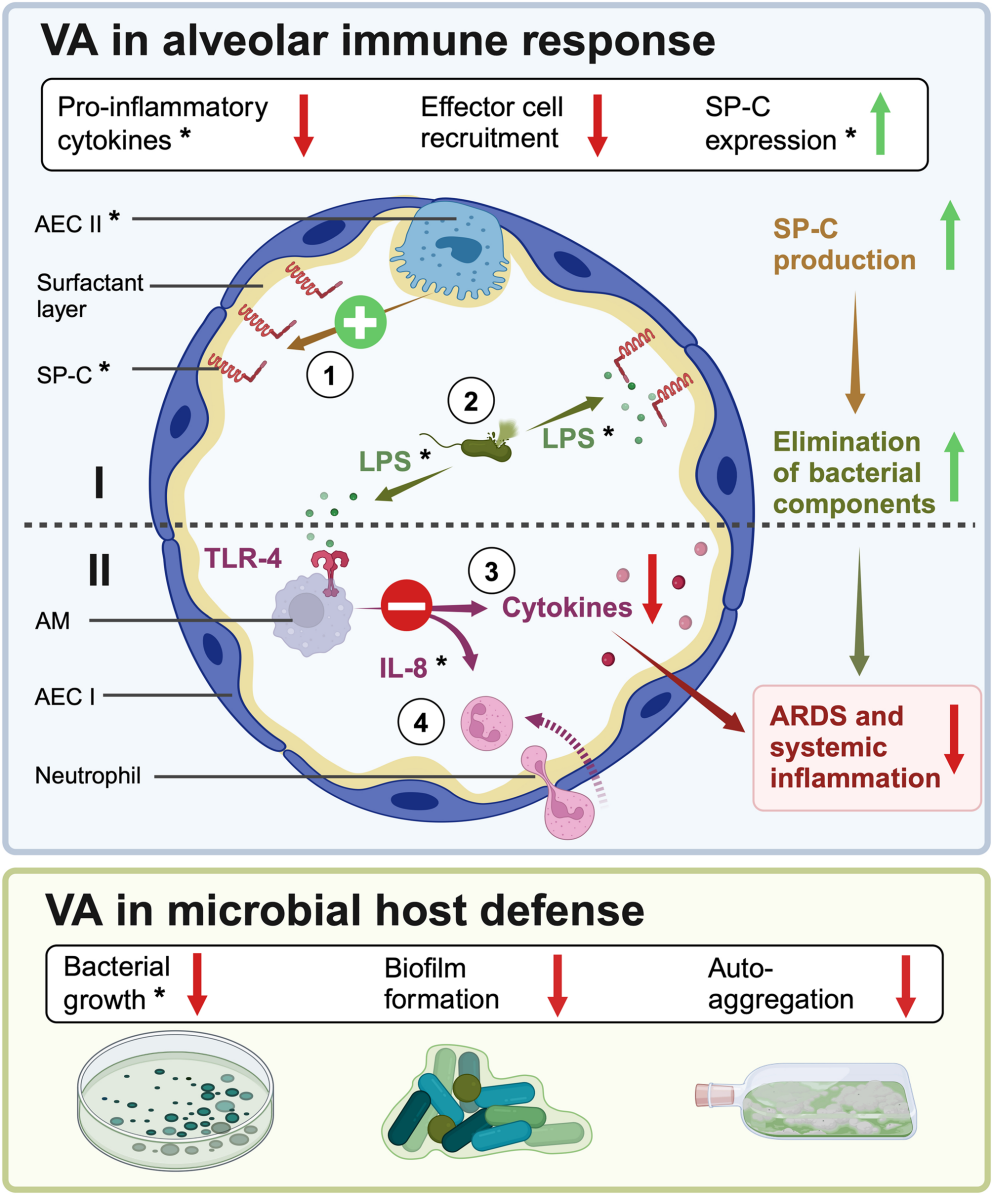
Synopsis of volatile anesthetics effect from literature and own findings. Based on the results of our experiments, the following mechanisms are proposed: Volatile anesthetics (VA), Sevoflurane (Sev, yellow) and Desflurane (Des, blue), exhibit both anti-inflammatory and anti-bacterial effects in our in vitro model of ARDS. Results from this in vitro study included in the overview figure are marked with an *. All other elements are derived from the following literature [6, 33, 42, 50]. (Upper panel) At the alveolar level, VA exert various effects: (**I**) They stimulate alveolar epithelial (AEC) Type II cells [1], leading to increased surfactant protein C (SP-C) production *. Since SP-C binds bacterial components like lipopolysaccharide (LPS) *, this enhances [2] the clearance of bacterial components. (**II**) Additionally, VAs inhibit [3] LPS-induced, toll-like receptor (TLR)−4-mediated production of pro-inflammatory cytokines * and [4] interleukin (IL)−8-driven recruitment of neutrophils. Together, these actions reduce both local and systemic inflammation in acute respiratory distress syndrome (ARDS). (Lower panel) VAs also directly affect bacterial growth *, biofilm formation, and autoaggregation, particularly in *P. aeruginosa*. AM: Alveolar macrophages

In summary, our experiments demonstrate that VA exert a distinct anti-bacterial influence on the growth of bacteria associated with hospital-acquired pneumonia. This effect is particularly pronounced for Gram-negative bacteria like *P. aeruginosa*, possibly due to their pronounced lipid bilayer, different autoaggregation activity and its inclination for biofilm formation (Fig. 5 lower panel). Further research should clarify the underlying mechanism of action for these observations.

Inflammatory processes are closely linked to the presence of pathogens and their byproducts, which can worsen bacterial pulmonal infections and contribute to the development of ARDS. One of the most significant mechanisms driving the pathophysiology of ARDS is the overproduction of cytokines [41]. One significant pro-inflammatory cytokine in this context is IL-8, which plays a crucial role in mediating the immune response as well as attracting neutrophils and other effector cells to sites of infection [42]. This plays a particular role in the initial phase of ARDS, which is characterized by neutrophilic alveolitis and disruption of the alveolar epithelial and endothelial barriers [43]. Moreover, elevated IL-8 levels have been identified as a predictive marker for ARDS progression and outcomes [44]. The induction of IL-8 production can be influenced by various factors, including the presence of bacteria and inflammatory stimuli such as LPS [45].

A549 cells have been shown to serve as a reliable in vitro model for the function of AEC II cells, as they form stable cultures as well as exhibit similar properties regarding cytokine and surfactant metabolism, particularly in long-term experiments [46]. In A549 lung epithelial cells, IL-8 is known to be reliably induced by LPS stimulation [47]. On this basis, we used IL-8 levels as surrogate for inflammatory response to LPS-stimulation. In accordance with the literature, we detected a significant increase of IL-8 after LPS exposure compared to baseline, particularly after 48 h. An increase in IL-8 concentration has been reported even in the absence of prior stimulation [48]. In our study, however, the observed rise in IL-8 levels following LPS stimulation was multifold compared to baseline conditions. Notably, when exposed to Sevoflurane and Desflurane, the effect of LPS on IL-8 levels was significantly diminished.

The anti-inflammatory effect of VA on IL-8 secretion observed in this study (Fig. 5, upper panel) is supported by previous data [13, 14]. Urner et al. [16] attributed this immunomodulatory effect to the fluorinated groups. In our study, the stronger effect in the Desflurane group could be a consequence of slight structural differences in fluorinated components compared to Sevoflurane [16]. In the lung, LPS induces inflammation through TLR-4 signaling, triggering pro-inflammatory cytokine production via the NFκB pathway in various cell types, including alveolar macrophages [49, 50]. Both in vivo and in vitro studies suggest that VA directly interact with the TLR-4/NFκB pathway, inhibiting pro-inflammatory cytokine production [51, 52]. VA may suppress alveolar macrophage activation and NF-κB signaling, reducing the release of cytokines like TNF-α and IL-8, thereby alleviating alveolar inflammation [50]. A direct modulation through β-adrenergic signaling on AECII has also been suggested [53].

Considering the critical role of LPS in activating TLR-4 signaling and promoting pro-inflammatory cytokine production, it is important to explore how these pathways relate to the regulation of related components of the innate immune system (Fig. 5.B). Surfactant proteins, particularly SP-A, have been associated with mitigating LPS-induced TLR-4 signaling, pathogen opsonization and phagocytosis [54, 55]. SP-C has mainly been recognized for its role in maintaining the proper structure of surfactant and stabilizing the surfactant film during breathing cycles [56]. However, next to the biophysical function of SP-C, recent studies revealed its contribution to immunomodulatory processes due to its anti-inflammatory properties [23, 57]. In vitro studies suggest that the underlying mechanisms involve an interaction between SP-C, cluster of differentiation (CD) 14, and LPS, as well as a direct binding and removal of LPS by SP-C [58–60]. Consistent with these findings, mice deficient in SP-A and SP-C demonstrate elevated levels of pro-inflammatory cytokines, display a hyper-inflammatory phenotype, and experience severe lung injury following pulmonary infections, which is associated with increased mortality rates [23, 55].

There are three reports on the effects of VA on surfactant proteins so far, all reporting an increased gene expression of the hydrophobic SP-B under Sevoflurane influence [14, 18, 61]. Araújo et al. [18] suggest, that a possible explanation for this effect includes reduced IL-6 as well as augmented transcription factors after Sevoflurane exposure, protecting against oxidative stress. This effect allows AECII cells to regain their function and capacity to synthesize surfactant proteins [18]. Similarly, our results show an increase in pro-SP-C protein expression — and to some extent in SP-A (Suppl. 3) — 24 h after exposure to VA. In Line with the protein expression data, SP-A mRNA expression also showed a moderate increase after 12 h of exposure to both Sevoflurane and Desflurane, followed by a return to baseline levels after 24 h. Given that surfactant synthesis is regulated by β-adrenergic agonists via a cyclic adenosine monophosphate (cAMP)-dependent promoter region [62, 63] a potential mechanism of action could involve direct stimulation of ß2-receptors by VA.

Interestingly, the observed increase in protein expression coincides with the reduction in VA-induced IL-8 release by A549 cells. Although no direct causality can be inferred, this finding suggests that surfactant proteins may contribute to the anti-inflammatory effects of VA on LPS-induced pro-inflammatory cytokine release in A549 cells (Fig. 5). Supporting this hypothesis, we observed a significant increase in pro-SP-C protein expression following co-stimulation with VA and LPS. While LPS alone is known to enhance SP-A and SP-D expression both in vivo and in vitro [64, 65] the here observed effect exceeds the SP-C stimulating effect of LPS alone. Studies reporting an increase in SP-A as well as SP-C protein and gene expression after LPS stimulation support these findings [46, 65].

While this study provides valuable insights into the potential mechanisms of anti-bacterial and anti-inflammatory actions of VA, it is important to recognize its limitations. Firstly, environmental variability, inconsistencies in bacterial cultures, and the anaerobic conditions within the Oxoid™ chamber can impact bacterial growth. We recognize that our choice of a fully anaerobic chamber (95% N₂, 5% CO₂) represents an extreme oxygen condition, even though briefly opening the culture flasks at each time point introduced small amounts of oxygen and, thus, prevented absolute anoxia. However, this was necessary to create a controlled environment, mimicking ARDS features such as limited gas exchange and acidic conditions from metabolic waste accumulation. Secondly, the duration, mode, and concentration of VA exposure are critical factors influencing the outcomes of this study. Thirdly, despite our efforts to simulate realistic conditions — such as replicating breathing cycles using mechanical ventilation with orbital shaking or ARDS-typical gas conditions — these methods cannot fully replicate the complex interactions and microenvironment of the alveolus. While we aimed to create conditions that were as clinically representative as possible, the subtle effects observed may have led to an underestimation of certain findings, such as the non-significant results for SP-A protein and mRNA expression. Nevertheless, our experimental findings may contribute to a deeper understanding of the complex mechanisms by which VA exert their anti-inflammatory and anti-bacterial effects (Fig. 5). Considering growing criticism surrounding the environmental impact of VA, their use should be more selectively targeted, leveraging their specific properties for patients with severe pulmonary infections and inflammation.

## Conclusion

In conclusion, prolonged anti-bacterial and anti-inflammatory effects of Sevoflurane and Desflurane include both the reduction of *P. aeruginosa* and *S. aureus* growth as well as the inhibition of LPS-induced chemokine release by A549 cells paralleled by an increase of surfactant protein expression, thereby potentially contributing to the favorable properties of VA in long-term sedation of ventilated patients with respiratory failure. If the proposed relationship between surfactant proteins and VA is confirmed in vivo, this pharmacological class might offer therapeutic benefits beyond sedation, although further research is required.

## Supplementary Information

Supplementary Material 1. Effect of VA on growth rate in log phase.

Supplementary Material 2. Effect of VA on A549 cells to VA over 48 on cell viability.

Supplementary Material 3. Effects of VA exposure on SP-A protein and SP-A mRNA expression under basal and LPS-stimulated conditions.

Supplementary Material 4. Full Western blots of fig. 4 and supplemental fig. 3

## Data Availability

The datasets used and/or analyzed during the current study are available from the corresponding author on reasonable request.

## Abbreviations

AEC II: Alveolar epithelial cells type II
ANOVA: Analysis of variance
ARDS: Acute respiratory distress syndrome
a.u.: arbitrary units
Ctrl: Control gas
CFU: Colony forming units
Des: Desflurane
*E. coli*: *Escherichia coli*
ELISA: Enzyme-linked immunosorbent assay
ICU: Intensive care unit
IL: Interleukin
LPS: Lipopolysaccharide
NF-kB: Nuclear factor kappa-light-chain-enhancer of activated B cells
OD_600_: Optical density at 600nm
*P. aeruginosa*: *Pseudomonas aeruginosa*
RPMI: Roswell Park Memorial Institute
*S. aureus*: *Staphylococcus aureus*
SDS-PAGE: Sodium dodecyl sulfate polyacrylamide gel electrophoresis
SEM: Standard error of the mean
Sev: Sevoflurane
Pro-SP-C: Propeptide of surfactant protein C
VA: Volatile anesthetics
