# Supplementary material for: Prolonged in vitro anti-bacterial, anti-inflammatory, and surfactant-promoting effects of volatile anesthetics
Source: BMC Pulm Med. 2025 Sep 9;25:425. doi: 10.1186/s12890-025-03849-w (PMC12421742; doi:10.1186/s12890-025-03849-w)

Supplement 4

Supplemental Figure 4 A-F: *Western Blot Analysis of ProSP-C, SP-A, and β-Actin expression over time and treatment conditions*

Complete Western blot images showing protein expression of the propeptide of surfactant protein C (ProSP-C, 21 kDa), surfactant protein A (SP-A, 35 kDa), and β-Actin (55 kDa) over 0 – 48 hours under different experimental conditions. Each blot image includes either 13 **(A, C, E)** or 12 **(B, D, F)** rows of protein samples. Protein size standards (PageRuler™, 10–180 kDa, Thermo Fisher) are depicted on the left axis. Panel **(A)** shows ProSP-C expression from 0 to 16 hours, including samples from control gas application (Ctrl) after 0h, 8h, and 16h; Sevoflurane (Sev) application for 8h and 16h; and Desflurane (Des) application for 8h and 16h, with additional lipopolysaccharide (+LPS) and no stimulation (Ø) blotted adjacent to each other. Panel **(B)** depicts ProSP-C expression from 24 to 48 hours, including samples from Ctrl 24h and 48h; Sev 24h and 48h; and Desflurane 24h and 48h, also with +LPS and Ø blotted adjacent. Similarly, SP-A expression is shown in panel **(C)** for 0 to 16 hours with the same sample arrangement as in panel (A), and in panel (D) for 24 to 48 hours with the same sample arrangement as in panel (B). Panels **(E)** and **(F)** present β-Actin expression for 0 to 16 hours and 24 to 48 hours, respectively, with identical sample layouts as described for the corresponding ProSP-C and SP-A panels.

A

ProSP-C 0 – 16 h

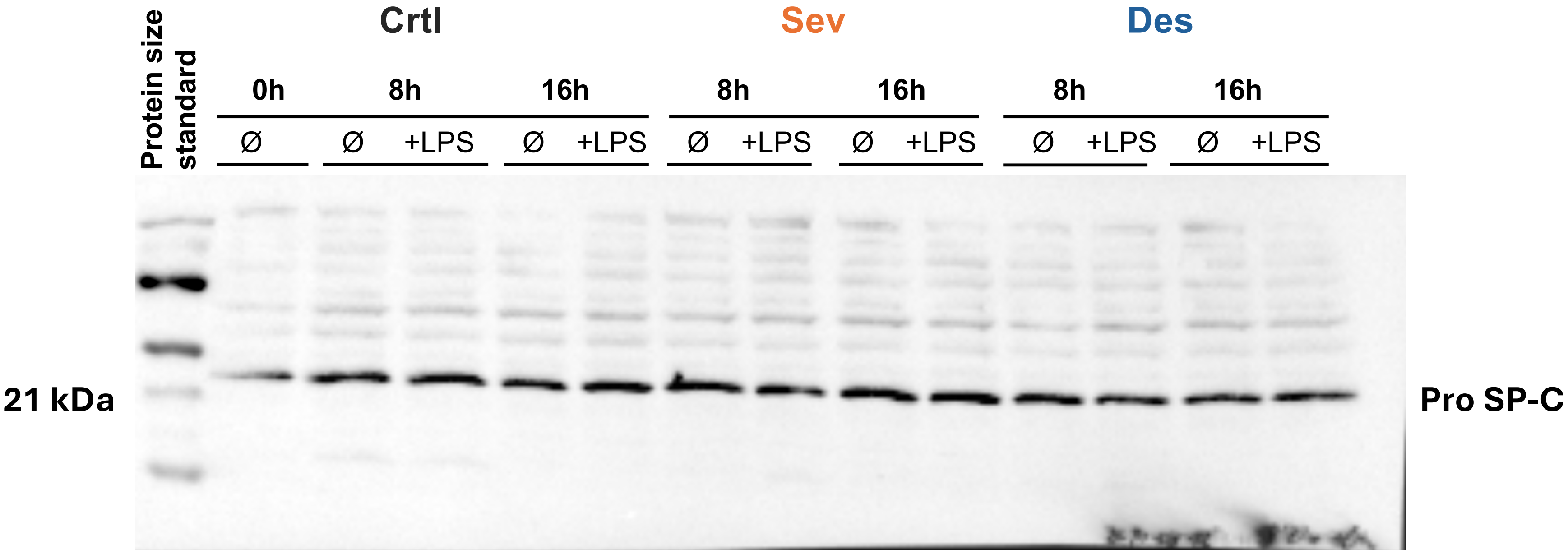

B

ProSP-C 24 – 48 h

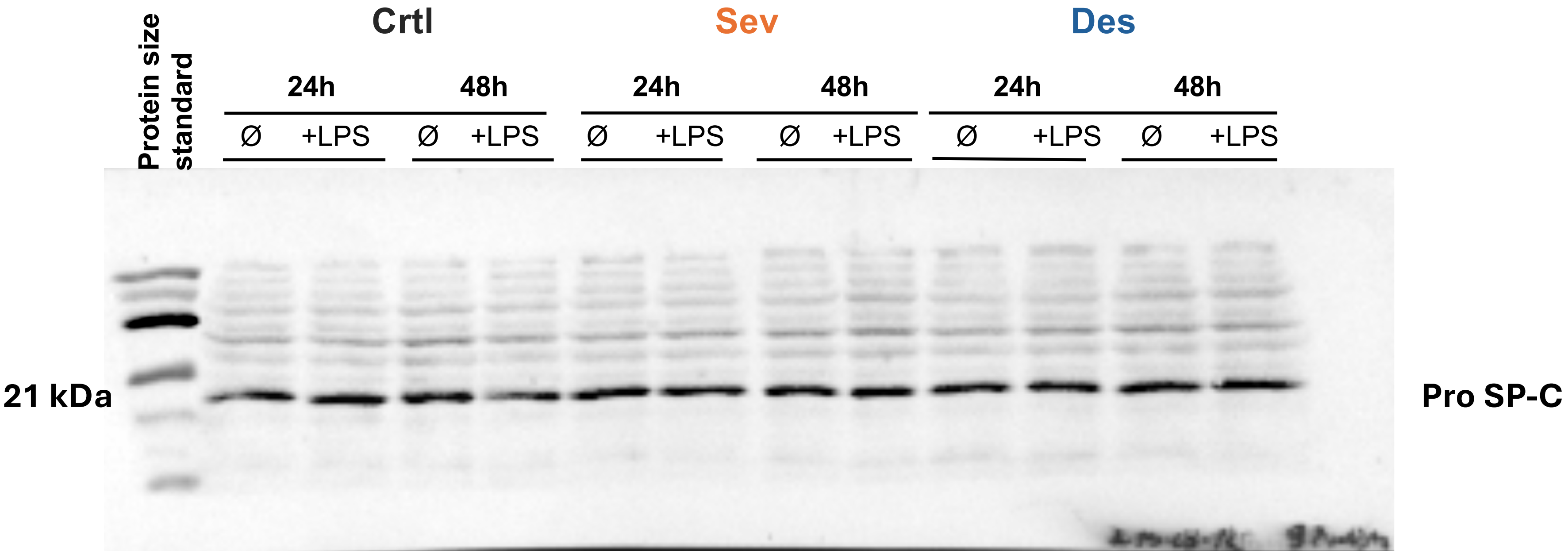

C

SP-A 0 – 16 h

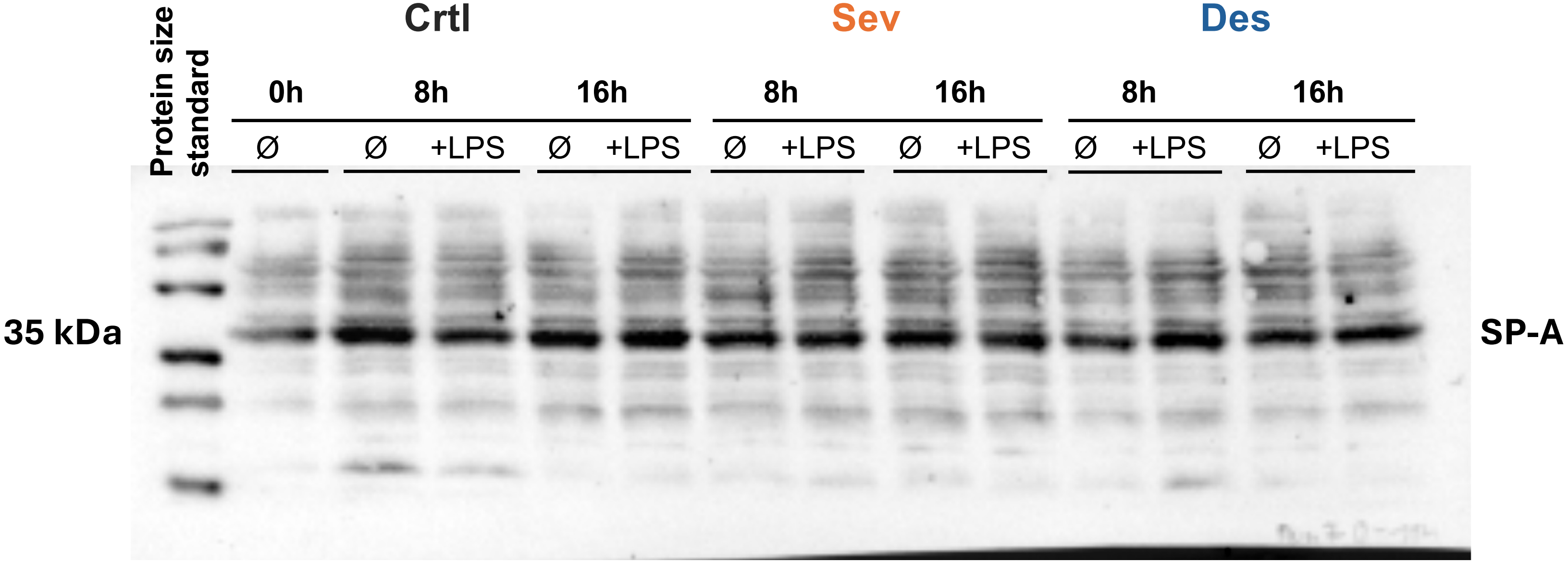

Protein size standard
Crtl
Sev
Des
0h
8h
16h
8h
16h
8h
16h
Ø
Ø
+LPS
Ø
+LPS
Ø
+LPS
Ø
+LPS
Ø
+LPS
Ø
+LPS
35 kDa
SP-A

D

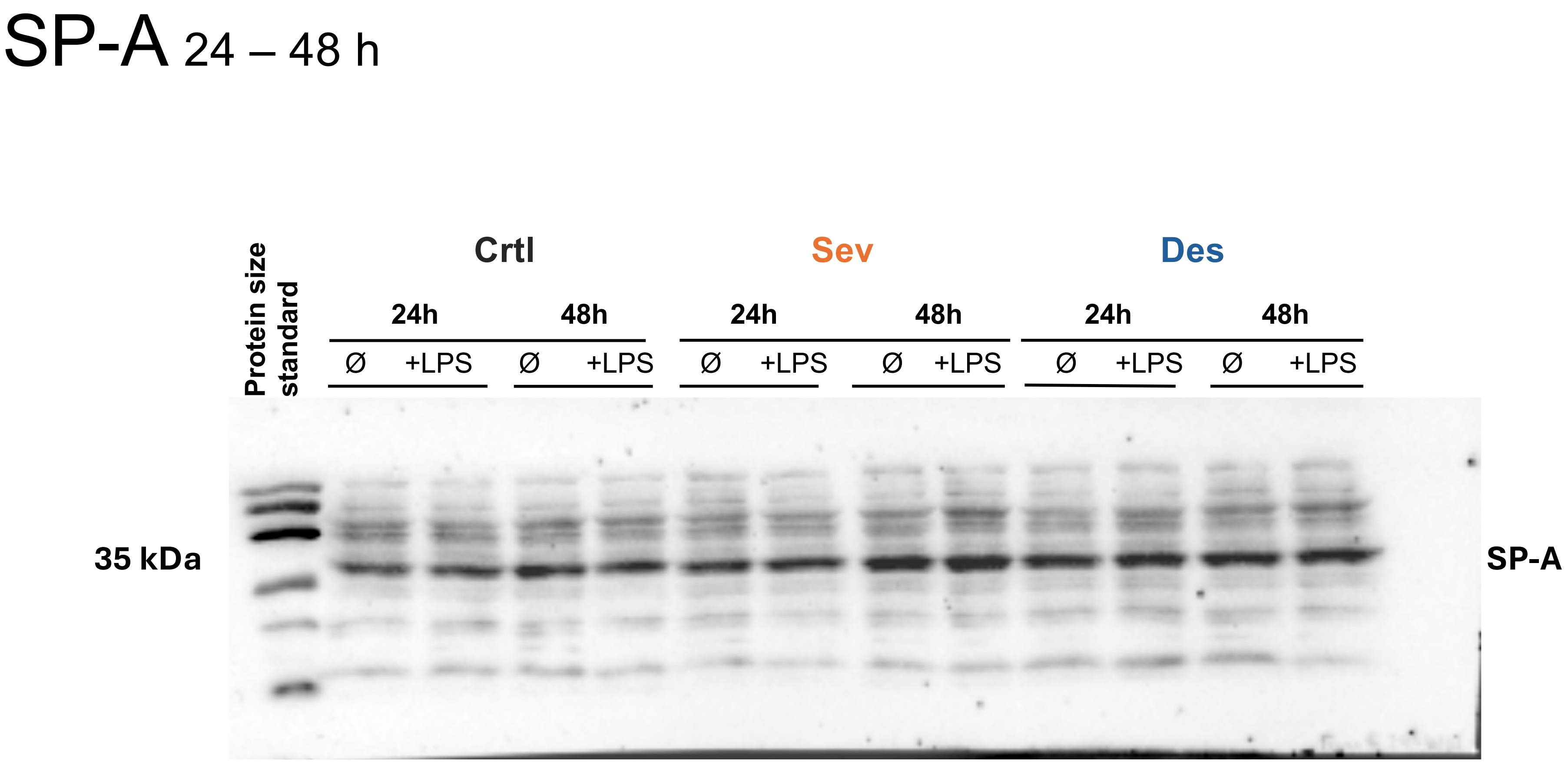
SP-A 24 – 48 h
Protein size standard
Crtl
Sev
Des
24h
48h
24h
48h
24h
48h
Ø
+LPS
Ø
+LPS
Ø
+LPS
Ø
+LPS
Ø
+LPS
Ø
+LPS
35 kDa
SP-A

E

β-Actin 0 – 16 h

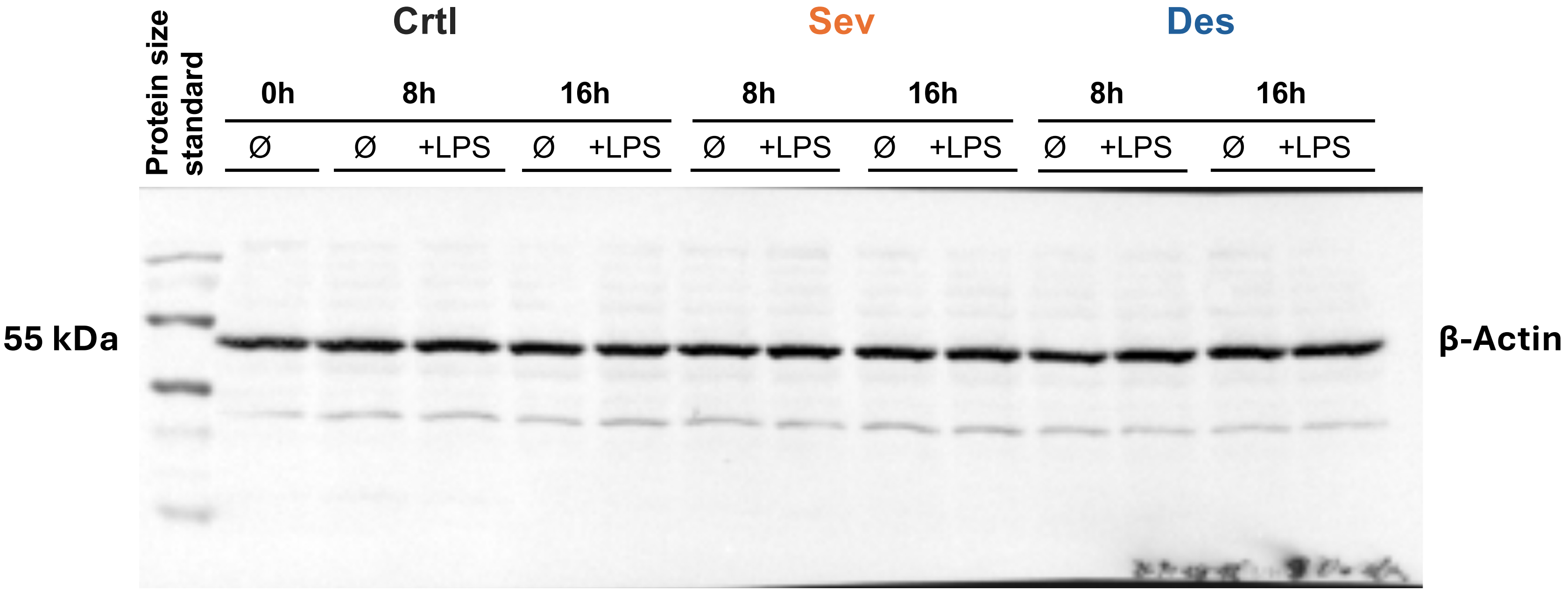

F

β-Actin 24 – 48 h

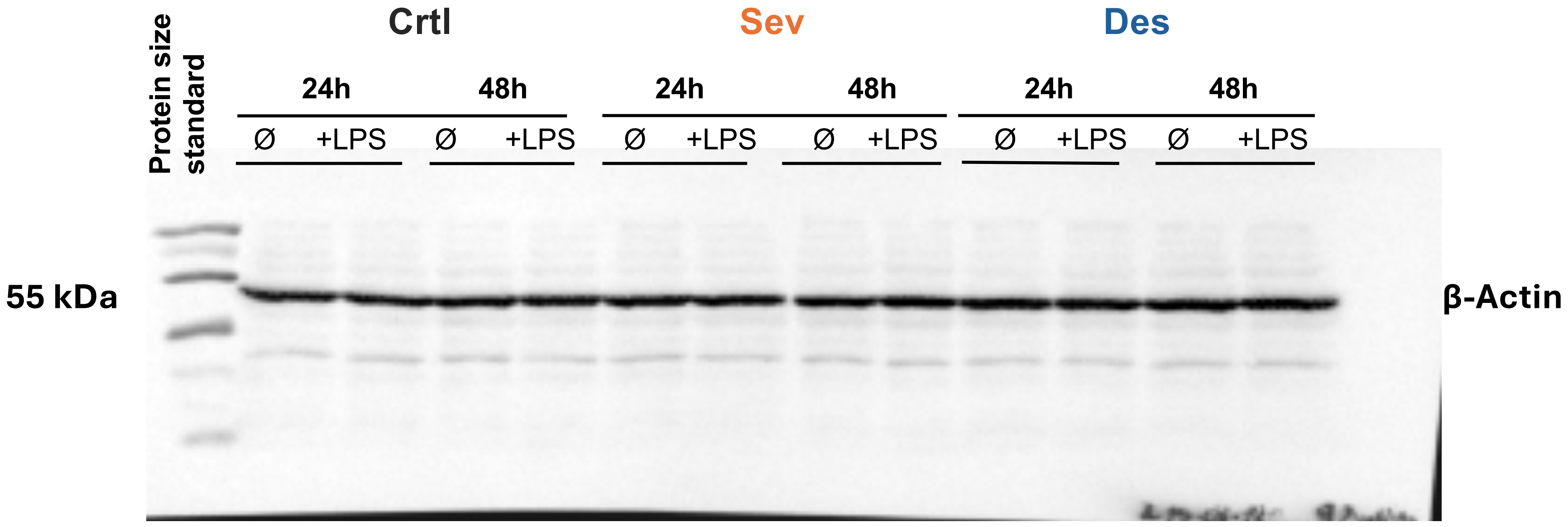

Supplement: Supplementary file 1 — Supplementary Material 1. Effect of VA on growth rate in log phase. [file 12890_2025_3849_MOESM1_ESM.pdf]
