# Supplementary material for: Prolonged in vitro anti-bacterial, anti-inflammatory, and surfactant-promoting effects of volatile anesthetics
Source: BMC Pulm Med. 2025 Sep 9;25:425. doi: 10.1186/s12890-025-03849-w (PMC12421742; doi:10.1186/s12890-025-03849-w)
Supplement: Supplementary file 2 — Supplementary Material 2. Effect of VA on A549 cells to VA over 48 on cell viability. [file 12890_2025_3849_MOESM2_ESM.pdf]

Supplement 1

Effect of volatile anesthetics on bacterial growth rate in *log phase*

Bacterial growth can be subdivided into different phases depending on the progression of the optical density at 600 nm wavelength (OD_{600}) changes. The *log phase* describes the period of unrestrained cell division with sufficient substrate and negligible metabolic waste (Suppl. Fig. 1.A). To determine the effect of volatile anesthetics (VA) on this crucial phase of bacterial cell division we compared the growth rate at the *log phase* under influence of both VA. None of the three bacterial strains was vulnerable to VA application or exhibited any changes in growth rate at *log phase* (Suppl. Fig. 1. B).

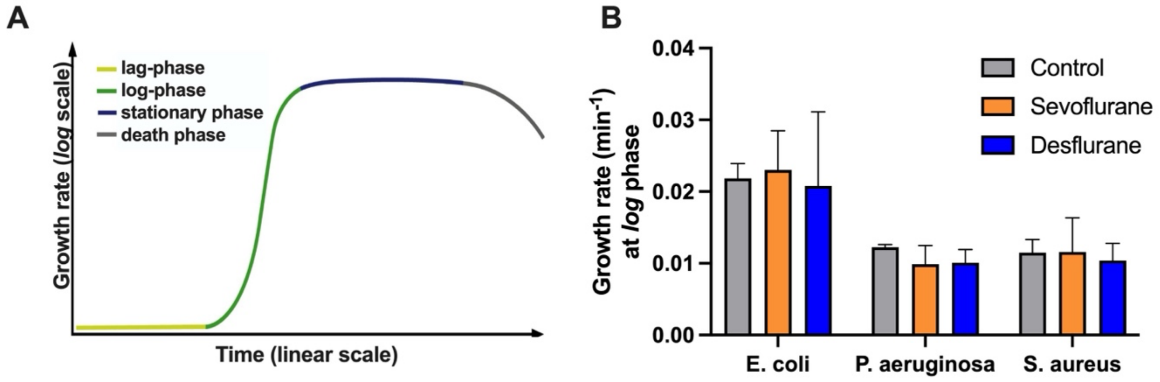

Suppl. Fig. 1: *Growth rate at log phase after exposure to volatile anesthetics (VA).*

(**A**) Bacterial growth can be subdivided into different phases derived from optical density at 600 nm wavelength (OD_{600}) measures. The *log phase* of bacterial growth is defined by optimal growth conditions resulting in exponential cell amplification. *Log phase* was determined in accordance to (Widdel, 2007). **(B)** Three bacterial strains were compared: two Gram-negative strains, *Pseudomonas aeruginosa* (*P. aeruginosa*), *Escherichia coli* (*E. coli*), and the Gram-positive strain, *Staphylococcus aureus* (*S. aureus*). Growth rate is depicted as min^{-1}. Data collection was performed after 6, 9, 12, 15, 18, 21, and 24 hours respectively. Ctrl: Control gas, Sevoflurane: Sev, Desflurane: Des. All results of are presented as mean +/- standard error of the mean (SEM) for n = 5 - 7.

Widdel, F. (2007). Theory and measurement of bacterial growth. Di Dalam Grundpraktikum Mikrobiologie, 4(11), 1–11.
