# Supplementary material for: Prolonged in vitro anti-bacterial, anti-inflammatory, and surfactant-promoting effects of volatile anesthetics
Source: BMC Pulm Med. 2025 Sep 9;25:425. doi: 10.1186/s12890-025-03849-w (PMC12421742; doi:10.1186/s12890-025-03849-w)
Supplement: Supplementary file 3 — Supplementary Material 3. Effects of VA exposure on SP-A protein and SP-A mRNA expression under basal and LPS-stimulated conditions. [file 12890_2025_3849_MOESM3_ESM.pdf]

## Supplement 3

Effects of volatile anesthetics exposure on SP-A protein expression under basal and LPS-stimulated conditions

To our knowledge, there are no studies analyzing the effects of VA on surfactant protein (SP)-A protein expression of A549 cells so far. Thus, we performed Western blot analysis of SP-A from A549 cells exposed to either control gas, Sevoflurane or Desflurane, respectively (Suppl. Fig. 3). The values (in arbitrary units [a.u.]) were then put into relation with control values to enable comparison over time. Desflurane exposure led to a slight increase after 24 hours (Suppl. Fig. 3.B, left). Challenging A549 cells additionally with LPS induced a slight increase in SP-A protein expression under control gas condition, corresponding to literature (Suppl. Fig. 3.B, right). To isolate the primary LPS-effect on surfactant protein expression under VA exposure as well as compare LPS-stimulated groups under VA exposure over time, a ratio for each time point was calculated by dividing each group value of the LPS-stimulated group (in arbitrary units [a.u.] for Ctrl + LPS, Sev + LPS and Des + LPS) by every unstimulated control group (Ctrl) value. The co-stimulatory effect of LPS and VA on SP-A protein expression was most prominent for Desflurane and peaked at 24 hours (Suppl. Fig. 3.B, right; Des + LPS, 24h vs 8 h: $p<0.05$). None of the other effects was considered significant.

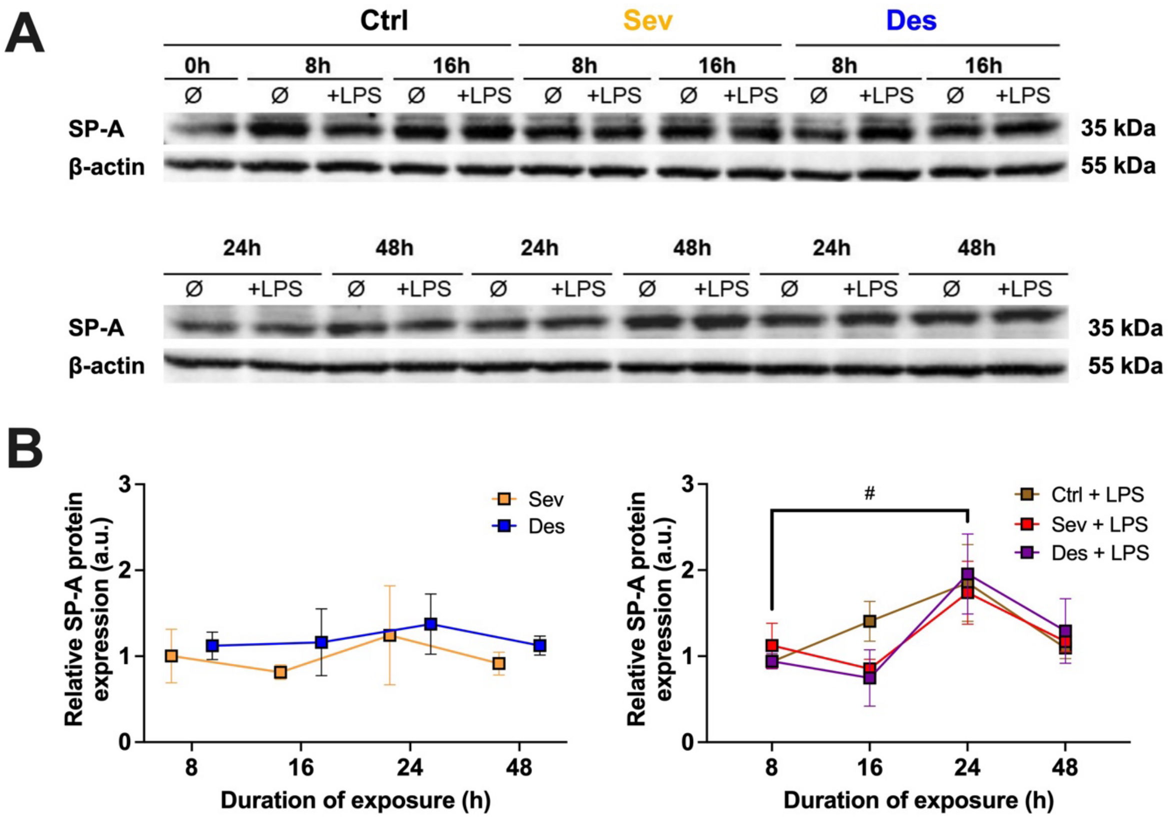


**Suppl. Fig. 3:** *Long-term effects (8, 16, 24, and 48 hours (h) exposure) of volatile anesthetics (VA) on surfactant protein (SP)-A protein expression by A549 cells under basal and lipopolysaccharide (LPS)-induced conditions.*

A549 cells (7.6 x$10^4$ cells/well) were treated with control gas (Ctrl; consisting of 95% room-air and 5% $CO_2$), 6.0% Desflurane (Des), or 2.1-2.2% Sevoflurane (Sev) in in the absence **(B left)** or presence of 1 µg/ml LPS **(B right)** for the times indicated. Equal amounts of cell lysates were subjected to SDS-PAGE and immunoblotted for SP-A and β-actin **(A)** (representative Western blot. Full blots are provided in Supplement 4). For comparison, the results presented in arbitrary units (a.u.) were put into relation to those of Ctrl at each time point. All results are presented as mean +/- standard error of the mean (SEM) for n = 5 – 6. Statistical significances are depicted as p<0.05*, Asterisks account for # = Des.

Effects of volatile anesthetics exposure on the relative SP-A mRNA expression after LPS stimulation

In contrast to the results of the propeptide of surfactant protein C (pro-SP-C), SP-A exhibits only a slight increase in relative protein expression in comparison to control conditions. Since protein analysis was performed from harvested cells and not culture medium, it is possible that due to prior excretion of SP-A we only identified a small portion of the total protein expressed and, thus, did not observe a significant effect.

Based on this assumption, we additionally analyzed the influence of VA on the expression of *messenger ribonucleic acid* (mRNA) by *real-time polymerase chain reaction* (RT-PCR) technique to capture also small increases in SP-A synthesis. First, we determined baseline expression of SP-A after Sevoflurane and Desflurane exposure only (Suppl. Fig. 4.A). Due to the small sample number (n = 2) time dependent effects could not be tested, however, a clear trend was visible for both Sevoflurane as well as Desflurane in A549 cells: the exposure to VA led to a 2-fold increase of SP-A mRNA expression compared to control gas, particularly for Sevoflurane.

Stimulating A549 cells with LPS is known to increase SP-A gene expression (Chuang et al., 2009). In our study, this effect was true, as the control gas infused- and LPS-stimulated cell culture showed a more than 2-fold increase after 4 hours and decreased consistently after that (Suppl. Fig. 4.B and C). However, under VA exposure, this effect seemed to be prolonged to 12h and increased, particularly for Sevoflurane. After 24 hours the expression of SP-A dropped to reference values regardless of VA or control gas exposure.

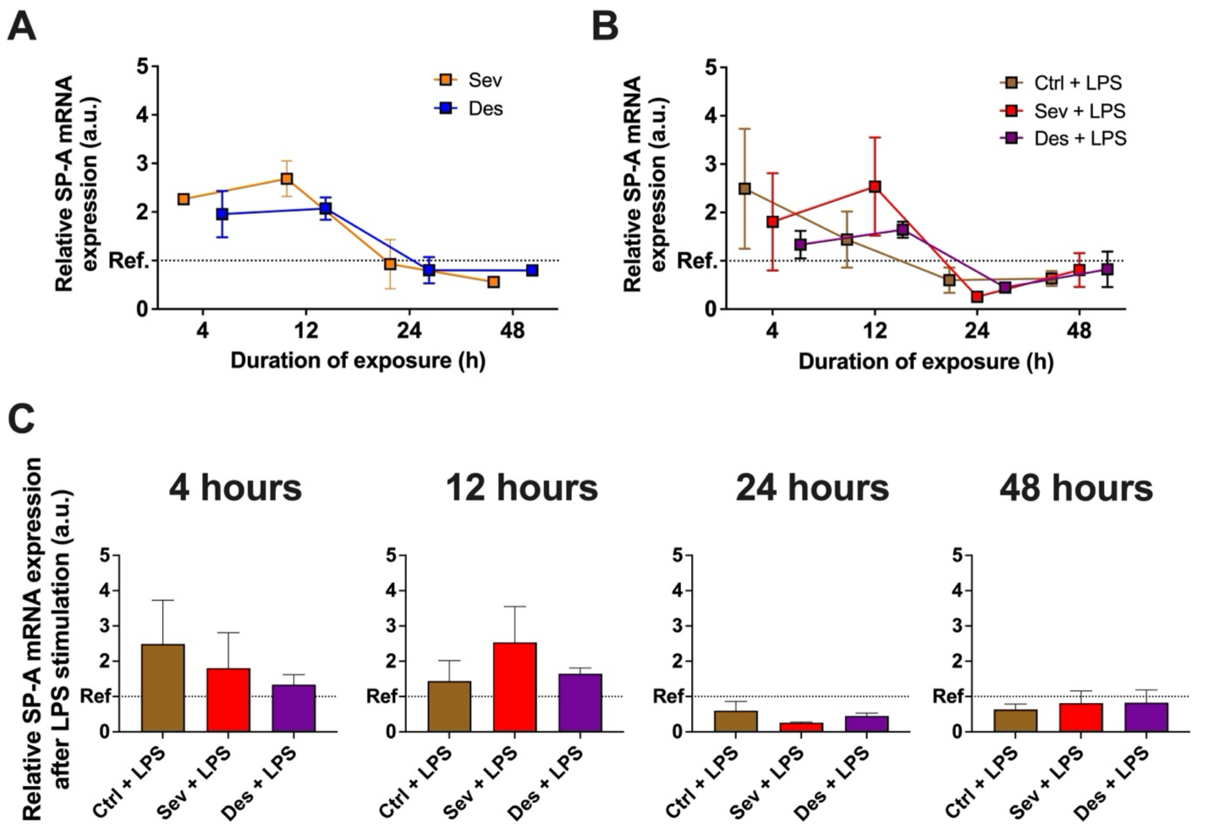


**Suppl. Fig. 4:** *Effect of prolonged (4, 12, 24, and 48 hours (h)) exposure of volatile anesthetics (VA) on surfactant protein (SP)-A mRNA expression by A549 cells under basal and lipopolysaccharide (LPS)-induced conditions.*

Analysis of mRNA expression was performed by RT-PCR. mRNA expression was calculated with the 2^ΔΔCT method with hu-TBP as housekeeping gene. Results were referenced to unstimulated Ctrl gas results (Ref) **(C)**. Relative mRNA expression is depicted in **(A)** for VA only conditions and in **(B, C)** under LPS stimulation. Crtl: Control gas consisting of 95% room-air and 5% $CO_2$, Sev: 2.1 - 2.2% Sevoflurane, Des: 6.0% Desflurane, LPS 1 µg/ml. All results are presented as mean +/- standard error of the mean (SEM) for n = 2.

*Supplemental Material & Methods*

Sample harvesting for cell viability testing was accomplished after 4, 12, 24, and 48 hours. RNA isolation, reverse transcription and RT-PCR: Total RNA was extracted from A549 cell lysates using the innuPREP RNA MINI Kit 2.0 (Analytik Jena AG, Jena, Germany) according to the manufacturer's instructions. The concentration of isolated RNA was determined with a spectrophotometer (DeNovix Inc., Wilmington, NC, USA). For each group a total amount of 1000 ng RNA was reverse transcribed into cDNA using deoxyribonucleic acids (Oligo dt 12-18, dNTP 10 mM; Thermo Fisher Scientific Inc.; incubation 5 Min. 65°C and cooling 5 Min. on ice) and DNA-polymerase (Second strand buffer, RNase OUTTM, SuperscriptTM, DTT; Thermo Fisher Scientific Inc; 60 Min. 50 °C and 15 Min. 70 °C). The primers were used as follows:

Human Surfactant-Protein A (hu-SP-A), (120 bp, Sigma-Aldrich Chemie GmbH)

sense 5´ CTGTCCCAAGGAATCCAGAG 3´,

antisense 5´ CCGTCTGAGTAGCGGAAGTC 3´

Human Tata-Box-Binding-Protein (hu-TBP), (69 bp, Roche Diagnostics GmbH, Mannheim, Germany)

sense 5´ GCTGGCCCATAGTGATCTTT 3´

antisense 5´ TCCTTGGGTTATCTTCACACG 3´

Samples were analyzed in a triple approach on the LightCycler platform (LightCycler® 480 Software, Version 1.5.1.62 SP3, Roche Diagnostics GmbH). The results were normalized to the housekeeping gene and calculated with the 2^ΔΔCT-Method (Livak & Schmittgen, 2001) referencing the control gas group without LPS stimulation (Ctrl).

---

Chuang, C.-Y., Chen, T.-L., & Chen, R.-M. (2009). Molecular mechanisms of lipopolysaccharide-caused induction of surfactant protein-A gene expression in human alveolar epithelial A549 cells. *Toxicology Letters*, *191*(2–3), 132–139. https://doi.org/10.1016/j.toxlet.2009.08.015

Livak, K. J., & Schmittgen, T. D. (2001). Analysis of Relative Gene Expression Data Using Real-Time Quantitative PCR and the 2−ΔΔCT Method. *Methods*, *25*(4), 402–408. https://doi.org/10.1006/meth.2001.1262
