# Supplementary material for: Prolonged in vitro anti-bacterial, anti-inflammatory, and surfactant-promoting effects of volatile anesthetics
Source: BMC Pulm Med. 2025 Sep 9;25:425. doi: 10.1186/s12890-025-03849-w (PMC12421742; doi:10.1186/s12890-025-03849-w)
Supplement: Supplementary file 4 — Supplementary Material 4. Full Western blots of fig. 4 and supplemental fig. 3 [file 12890_2025_3849_MOESM4_ESM.pdf]

Supplement 2

Cell viability over 48 hours throughout exposure of A549 cells to volatile anesthetics

We performed cell viability testing to determine toxicity effects of volatile anesthetics (VA) on A549 cells over time (8, 16, 24 and 48 hours). Cell viability was assessed by Erythrosin B staining and subsequent manual counting. The lowest cell viability was detected for Sevoflurane treatment (90% [87 – 96], averaged over all time points). Over 90% of cells remained viable in all groups, excluding a pronounced toxicity effect of VA on A549 cells (Suppl. Fig. 2.A). This result was also reproducible in the lipopolysaccharide (LPS) group, despite potential toxic effects of LPS on cell cultures. Here, Sevoflurane plus LPS showed the lowest value (91,5% [88 – 93]; Suppl. Fig. 2.B).

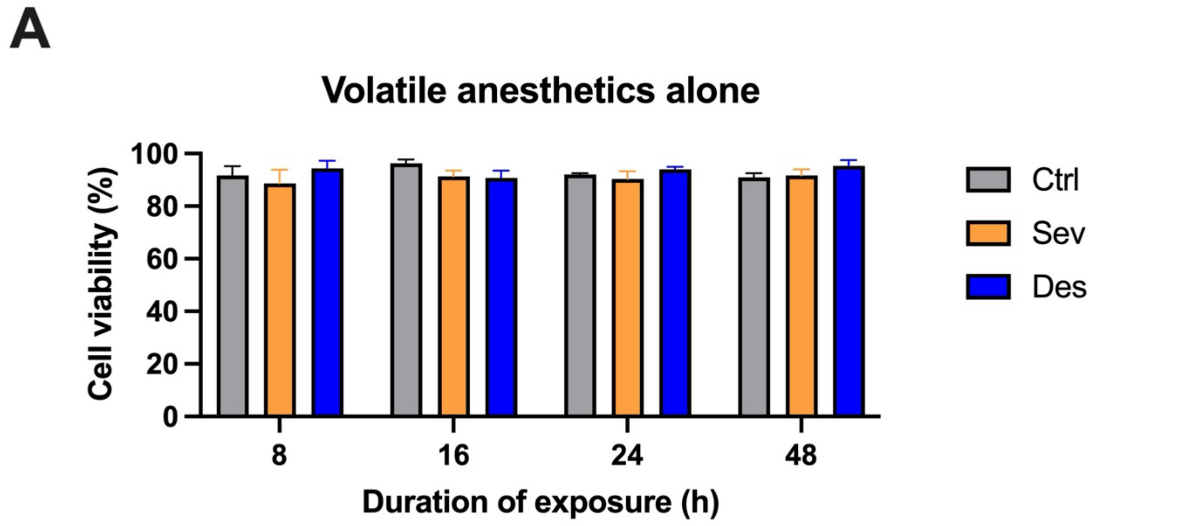

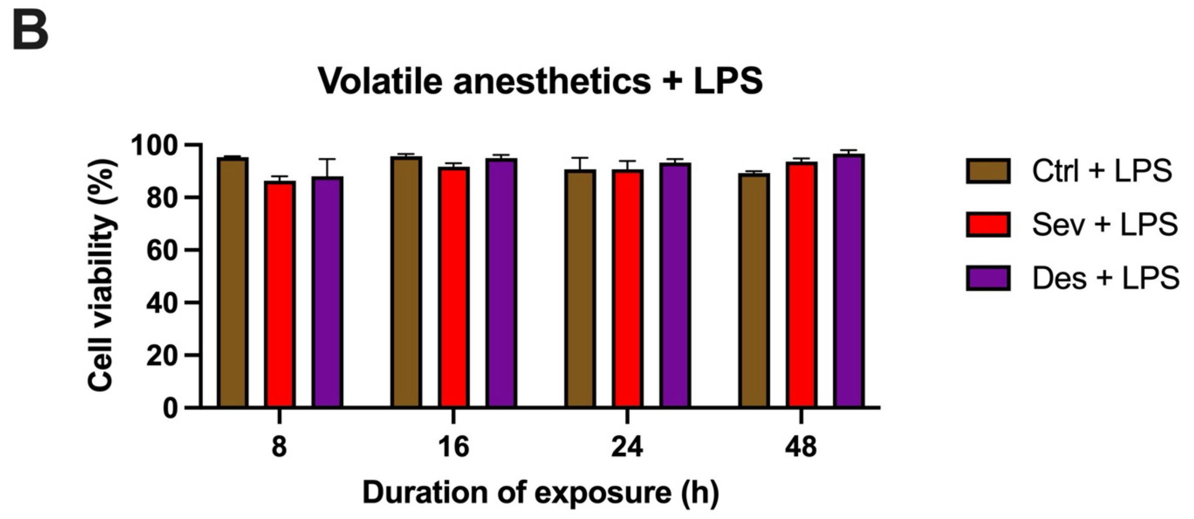

Suppl. Fig. 2: *Long-term effects (8, 16, 24 and 48 hours (h) exposure) of volatile anesthetics (VA) on the viability of A549 cells under basal and lipopolysaccharide (LPS)-induced conditions.* **(A)** Viability testing was performed for exposure to VA alone (Control gas (Ctrl; consisting of 95% room-air and 5% CO_2), 2.1 – 2.2% Sevoflurane (Sev), 6.0% Desflurane (Des)) and **(B)** exposure to VA plus 1 µg/ml LPS. Quantification of viability (% of total) in both conditions was achieved by manual cell count after Erythrosin B staining. Results are presented as mean +/- standard error of the mean (SEM) for n = 3 per time point.
